# Insulinotropic Potential of *Moxifloxacin* and *Gemifloxacin:* An In Vivo Rabbits Model Study Followed by Randomized Phase I Clinical Trial

**DOI:** 10.3390/antibiotics11020148

**Published:** 2022-01-24

**Authors:** Abid Ullah, Shujaat Ahmad, Niaz Ali, Shafiq Ur Rahman, Haya Hussain, Saad Alghamdi, Mazen Almehmadi, Anas S. Dablool, Azzah M. Bannunah, Syeda Hajira Bukhari, Feras Almarshad

**Affiliations:** 1 Department of Pharmacy, Shaheed Benazir Bhutto University, Sheringal, Dir Upper 18000, KPK, Pakistan; abid@sbbu.edu.pk (A.U.); shafiq@sbbu.edu.pk (S.U.R.); haya@sbbu.edu.pk (H.H.); 2 Department of Pharmacology, Institute of Basic Medical Sciences, Khyber Medical University, Peshawar 25100, KPK, Pakistan; syedhajirabukhari@gmail.com; 3 Department of Pharmacology, College of Medicine, Shaqra University, Shaqra 11961, Saudi Arabia; 4 Laboratory Medicine Department, Faculty of Applied Medical Sciences, Umm Al-Qura University, Makkah 24211, Saudi Arabia; ssalghamdi@uqu.edu.sa; 5 Department of Clinical Laboratory Sciences, College of Applied Medical Sciences, Taif University, Taif 21944, Saudi Arabia; dr.mazen.ma@gmail.com; 6 Department of Public Health, Health Sciences College at Al-Leith, Umm Al-Qura University, Makkah 24211, Saudi Arabia; asdablool@uqu.edu.sa; 7 Department of Basic Sciences, Common First Year Deanship, Umm Al-Qura University, Makkah 24211, Saudi Arabia; ambannunah@uqu.edu.sa; 8 Department of Medicine, College of Medicine, Shaqra University, Shaqra 11961, Saudi Arabia; Falmarshad@su.edu.sa

**Keywords:** *Moxifloxacin*, *Gemifloxacin*, insulin, hypoglycemia, glycaemia, dysglycemia, *Glibenclamide*, in vivo rabbits model, phase I clinical trial

## Abstract

Fluoroquinolones (FQs) have been reported to cause dysglycemia in both diabetic and non-diabetic patients. However, diabetic patients are usually on polypharmacy, so we cannot attribute the dysglycemia specifically to FQs. To answer the question as to whether *Moxifloxacin* and *Gemifloxacin* influence blood glucose levels and serum insulin levels or otherwise, rabbits were used as experimental animals in an in vivo model followed by a phase I randomized clinical trial in euglycemic healthy volunteers. The effects on the serum insulin and blood glucose levels in the *Moxifloxacin* and *Gemifloxacin* treated groups were, respectively, determined on the fifth day in both the in-vivo rabbits model and in the test subjects of the phase I clinical trial. The effects of these drugs were also checked on the histomorphology of the pancreas in the rabbits. The findings of our study suggest that *Moxifloxacin* and *Gemifloxacin* significantly (*p* < 0.05) reduced the blood glucose levels via a subsequent significant shift in the serum insulin levels both in the in vivo animal model and in the test subjects of the phase I clinical trial. No prominent effects on the beta cells histomorphology were noted in this study. *Moxifloxacin* showed a more significant effect than *Gemifloxacin*. The insulinotropic effect was comparable to the effect of *Glibenclamide*. It is concluded that *Moxifloxacin* and *Gemifloxacin* have a significant blood glucose lowering effect mediated through insulinotropic action. (Clinical Trials.gov identifier: NCT04692623).

## 1. Introduction

Fluoroquinolones (FQs) belong to a class of broad-spectrum antibiotics used to kill various microorganisms [1,2]. During the last two decades, newer fluoroquinolones have been introduced into the market [3]. These newer FQs were synthesized in the laboratory from *Nalidixic acid*, which was synthesized as a byproduct of antimalarial chloroquine [4]. Among these, the older *Ciprofloxacin* and *Norfloxacin* are the drugs of choice for gastro and urinary tract infections. Newly introduced *Moxifloxacin* is popular due to its better potency against infections of the upper and lower respiratory tract [5]. There are reports for antimitotic and antimalarial activities along with the antibacterial activities of the FQs [6]. Some serious adverse effects of FQs have also been reported besides its excellent therapeutic profile [7]. Many FQs have been recalled and banned due to causing severe adverse drug reactions. These adverse effects have badly affected the therapeutic significance of FQs. These adverse effects have also raised serious concerns regarding their safety profile [8]. Among these, tendinitis, cardiotoxicity, and dysglycemia are some of the top serious adverse effects noted with the use of FQs [9]. *Trovofloxacin* and *Sparfloxacin* were withdrawn from the market due to severe hepatotoxicity, phototoxicity, and cardiotoxicity [10]. *Temofloxacin* and *Levofloxacin* have limited use nowadays due to their phototoxic effects [11]. Previously, *Enoxacin*, *Lomefloxacin*, and *Norfloxacin* were causing severe hypoglycemia. *Levofloxacin* and *Gatifloxacin* have been reported for both hypoglycemia and hyperglycemia [12]. *Gatifloxacin*, due to its high rate of causing severe hyperglycemia, was withdrawn from the market [13]. Three marketed fluoroquinolones, namely *Gatifloxacin*, *Levofloxacin*, and *Moxifloxacin*, were placed in the Metabolic and Nutritional Disorders category by the Canadian Adverse Drug Reaction Monitoring Program (CADRMP) [14]. *Moxifloxacin* was approved by the US FDA in 1999 [15]. *Moxifloxacin* has also been reported for changes in the blood glucose homeostasis and abnormalities with both hypoglycemic and hyperglycemic effects [16]. Reports regarding the hyperglycemic and hypoglycemic effects of FQs on blood glucose levels remain controversial, so, in the current study, the newer fourth generation fluoroquinolones (*Moxifloxacin* and *Gemifloxacin*) were tested in a controlled in vivo environment (euglycemic rabbits), followed by their translation in a randomized phase I clinical trial in the healthy human volunteers (euglycemic) to identify their dysglycemic effects.

## 2. Results

### 2.1. Moxifloxacin and Gemifloxacin Effects on Blood Glucose and Serum Insulin Levels in In Vivo Rabbits Model

The concept of the possible effects on the release of insulin with a subsequent shift in the blood glucose levels in the working pancreas of euglycemic rabbits is due to activation of ATP sensitive K channels; hence, we designed the current in vivo rabbits model to translate the possible beta cells’ cytotropic effects in the working pancreas. The blood glucose levels of the experimental animals (treated and untreated groups) on the first day (as baseline) and on day five were determined. The values of blood glucose level (mg/mL) were shown in Table 1. The blood glucose levels were reduced significantly (*p* < 0.05) in the test drug treated groups versus the respective untreated (negative control) and positive control groups (standard *Glibenclamide*) (Table 1). Similarly, a surge in serum insulin levels on day five of all the drug treated and untreated groups was observed, as shown in Table 2. The serum insulin levels were significantly (*p* < 0.0001) increased in the *Moxifloxacin* treated group as compared to the *Gemifloxacin* treated group (*p* < 0.05). This shows that the effect of *Moxifloxacin* was more significant than the effect of *Gemifloxacin.*

**Figure 1:**
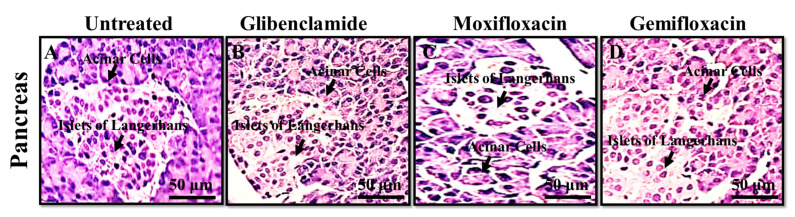
Sections (5µ) of rabbit pancreas using H&E staining (**A**) untreated, (**B**) *Glibenclamide*, (**C**) *Moxifloxacin*, (**D**) *Gemifloxacin* treated groups showing normal islets of Langerhans with no histomorphological changes.

No significant changes in the cellularity and density of the beta cells of the pancreas were noted in our present study in all the treated groups, as shown in Figure 1.

#### Tissues Histomorphology

No significant changes in the cellularity and density of the beta cells of the pancreas were observed in all the treated groups as we administered the test (*Moxifloxacin*, *Gemifloxacin*) and standard (*Glibenclamide)* drugs for 5 days (Figure 1).

### 2.2. Moxifloxacin and Gemifloxacin Effects on Blood Glucose and Serum Insulin Levels in Healthy Volunteers in Phase I Clinical Trial

In the phase I clinical trials, sixty-five healthy volunteers were screened for eligibility. Five subjects did not fulfill the inclusion criteria. The participants’ demographic information and baseline clinical characteristics are shown in Table 3.

**Figure 2:**
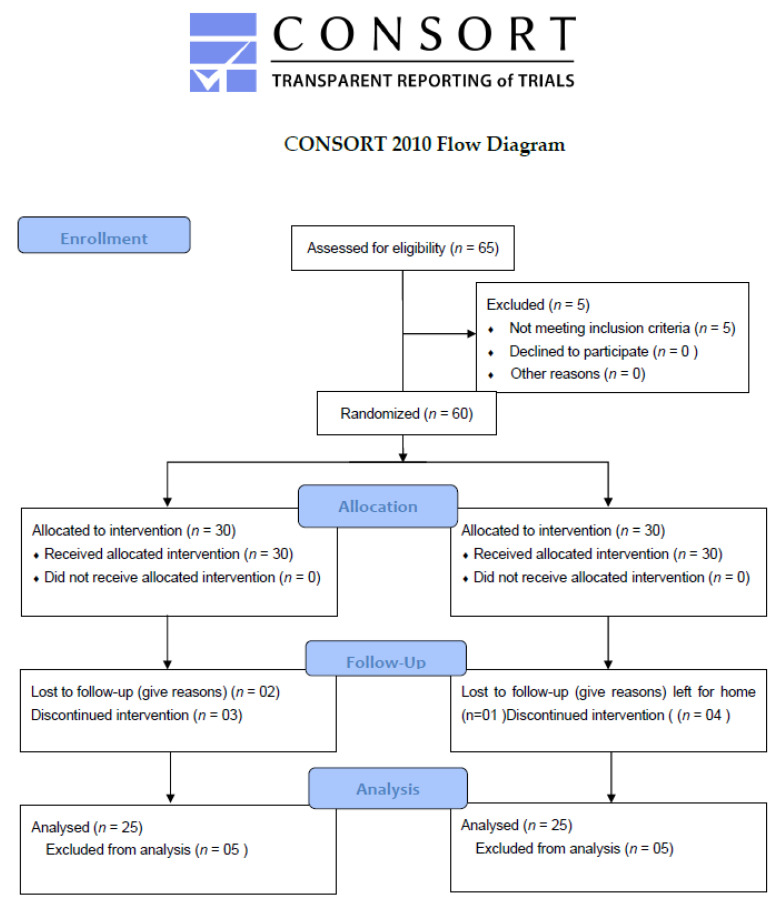
Clinical trial profile.

Sixty volunteers were selected and were enrolled in the trial (Figure 2, Clinical trial profile). 

The mean blood glucose levels were significantly decreased (*p* < 0.0001) with *Moxifloxacin* as compared to the respective baseline in the *Moxifloxacin* treated group. However, a relatively less significant effect (*p* ˂ 0.05) was achieved in the *Gemifloxacin* treated group in healthy volunteers. The serum insulin level was increased more significantly (*p* ˂ 0.0001) in the *Moxifloxacin* treated group, and a less significant effect (*p* ˂ 0.05) was observed in the *Gemifloxacin* treated group. Initially, the results were reproduced in the rabbits animal model studies, where more significant effects for *Moxifloxacin* were noted as compared to *Gemifloxacin*. The results of the animal model study reveal that *Moxifloxacin* and *Gemifloxacin* significantly (*p* < 0.05) reduced blood glucose levels with a significant (*p* < 0.05) surge in insulin level. Moreover, we observed significant increases in the serum insulin level in both the *Moxifloxacin* and *Gemifloxacin* treated groups (*p* < 0.05). Hence, we report that the *Moxifloxacin* and *Gemifloxacin* produced significant blood glucose lowering effects via release of insulin, with a more significant effect for *Moxifloxacin* than *Gemifloxacin*.

**Figure 3:**
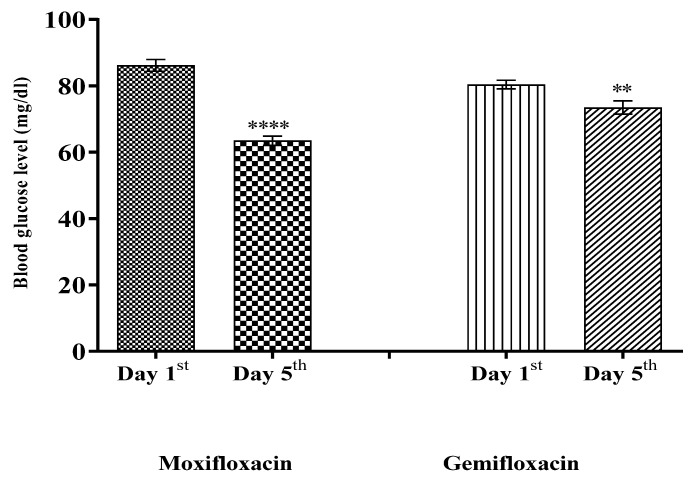
Comparison of blood glucose levels on day 1st and on day 5th of healthy volunteers treated with *Moxifloxacin* and *Gemifloxacin*, respectively. Significant differences in blood glucose levels were noted on day 5th; *p*-values ˂ 0.0001 and ˂0.05 were expressed as **** and ** respectively. One-way (ANOVA) test followed by column statistics and Wilcoxon signed rank test analysis were applied.

**Figure 4:**
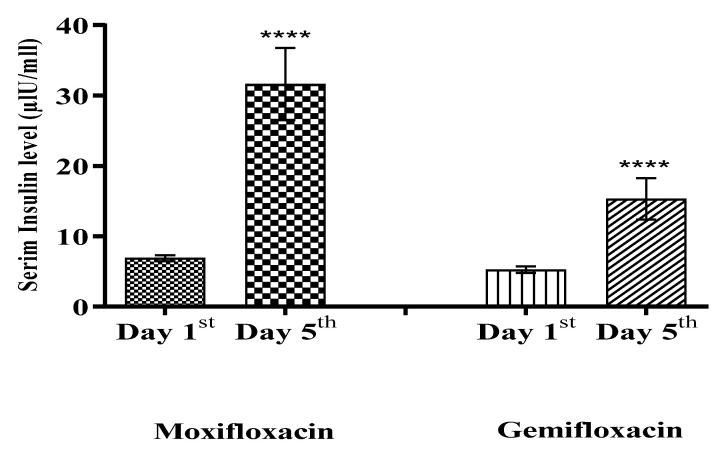
Comparison of serum insulin levels on day 1st and on day 5th of healthy volunteers treated with *Moxifloxacin* and *Gemifloxacin*, respectively. Significant surges in serum insulin levels in both treated groups were noted on day 5th. One-way (ANOVA) test followed by column statistics and Wilcoxon signed rank test analysis were applied. *p*-values ˂ 0.0001 were expressed as ****.

In the healthy volunteers of the clinical trial, the *Moxifloxacin* and *Gemifloxacin* effects on blood glucose and serum insulin levels are shown in Figure 3 and Figure 4, respectively. 

*Moxifloxacin* and *Gemifloxacin* caused a significant reduction in the blood glucose level, with a subsequent shift in the insulin level in the experimental rabbit model, and these are also translated in the phase I clinical trial. This study reports that *Moxifloxacin* and *Gemifloxacin* produced significant blood glucose lowering effects via release of insulin.

## 3. Discussions

In this study, *Moxifloxacin* and *Gemifloxacin* best describe the possible insulinotropic effect for the decrease in blood glucose levels. We observed a classical surge in the serum insulin levels with a subsequent decrease in the blood glucose levels. Though there are reports of dysglycemia regarding the use of fluoroquinolones in treating different ailments with quinolones, previous case studies reported that quinolones induce dysglycemia [17,18,19,20]. However, there are also reports of hyperglycemia with *Moxifloxacin*, *Gatifloxacin*, and *levofloxacin*. *Moxifloxacin* has been related with dysglycemia, but the clear picture of hypoglycemia and hyperglycemia has not yet been reported in the published studies [15,21].

In this study, group “A” was kept as the untreated group and was fed with fresh water and food only. The results of our studies showed that, in the controlled environment, these factors (food and fresh water) had no significant effect on the blood glucose and serum insulin levels. This confirms the hypoglycemic potential of the current fluoroquinolones (*Moxifloxacin* and *Gemifloxacin*), similar to that produced by *Glibenclamide*. It is evident from case reports and other studies that fluoroquinolones may cause dysglycemia, i.e., hypoglycemia and or hyperglycemia. In hyperglycemia, more insulin secretion is related to pancreatic vacuolation with concomitant use of quinolones [22]. In this study, *Moxifloxacin* and *Gemifloxacin* significantly decreased blood glucose levels, with proportionate surges in the serum insulin levels in euglycemic rabbits as well as in healthy euglycemic volunteers, which implies an insulinotropic effect of *Moxifloxacin* and *Gemifloxacin*.

On the other hand, high blood glucose levels also stimulate the pancreatic beta cells and thus ultimately cause insulin secretion [23]. It is pertinent to mention that the release of insulin was increased versus the baseline insulin levels. A high concentration of blood glucose level causes an increase in ATP and ADP levels, which causes depolarization of pancreatic beta cells. This further causes opening of voltage gated (vg) calcium channels that allow the influx of calcium in the β cells, which ultimately causes insulin release from the working pancreas [20,24]. This mechanism is true for the blood glucose lowering effect of *Glibenclamide* used as a standard drug. However, regarding *Moxifloxacin* and *Gemifloxacin*, this mechanism is yet to be explored. However, it is clear that *Moxifloxacin* and *Gemifloxacin* caused insulin surges that produced blood glucose lowering effects, although there are reports that that hypoglycemic effect of fluoroquinolones is due to activation of ATP sensitive K channels in pancreatic beta cells [25,26]. Additionally, we translated that *Moxifloxacin* and *Gemifloxacin* produced a fall in blood glucose levels.

*Glibenclamide* causes prolonged hypoglycemic effects [27] by binding to SUR1 and SUR2 (sulfonylurea receptors) in pancreatic beta cells, causing activation of the ATP-sensitive potassium channels [28]. This further causes calcium influx and insulin secretion via the opening of voltage gated calcium channels, and a hypoglycemic effect is achieved. Similar results were observed in the *Moxifloxacin* and *Gemifloxacin* treated groups [29].

In order to discover possible histological changes in gross morphology and histology, on the completion of the experiment, all the rabbits were slaughtered and their pancreases were excised, as per Mir et al., 2013 [30]. The beta cells density and cellularity in all the treated and untreated groups apparently remained the same and no prominent changes were observed, which still needs to be explained.

Thus, our findings now translate the already existing case reports of hypoglycemia associated with the use of *Moxifloxacin* [31]. Previous studies report that fluoroquinolones cause hypoglycemia when used concomitantly with sulfonylurea [20]. However, the newer quinolones are comparably potent to decrease the blood glucose levels via an increased surge in the release of serum insulin. Thus, the use of the current quinolones carries the risk of developing hypoglycemia amongst the diabetic patients who are already controlled on sulfonylureas and require antibiotic therapy.

In our present study, we only determined the effect of these drugs on blood glucose via any surge in the serum insulin level. Thus, blood samples were taken and respective surges in insulin levels were noted. It is not necessary that every surge in insulin shall be associated with hypoglycemia as there are other compensatory mechanisms, such as the secondary messenger system, i.e., secretion of glucagon, sympathomimetic activity (secretion of epinephrine), and a glycogenolysis-like effect, which were not the domain of our study at this stage and require further studies to identify the underlying mechanism in euglycemics (working pancreas).

## 4. Materials and Methods

### 4.1. In Vivo Animal Model Studies

#### 4.1.1. Drugs and Preparation of Drugs Solutions

All the drugs used in this research were of standard pharmaceutical grade. *Moxifloxacin*, *Gemifloxacin*, and *Glibenclamide* were, respectively, purchased from Getz, Sami, and Sanofi Aventis pharmaceutical companies. *Moxifloxacin*, *Gemifloxacin*, and *Glibenclamide* were given on the basis of average body weights calculated from normal adult normal doses. *Moxifloxacin* and *Gemifloxacin* salts were soluble in water as both are available in salt form. *Moxifloxacin Hydrochloride* and *Gemifloxacin mesylate* (5 mg/kg body weight) were solubilized in water for injection, while *Glibenclamide* (0.2 mg/kg) was given in hard gelatin capsule form orally as it was insoluble in water. Starch was used as excipient by using geometric dilution and extemporaneously filling of the capsules. The content uniformity of active ingredient was assured as per ranges mentioned in the BP 2011 Appendix XIIC. 

Four groups had 6 rabbits (*n* = 6) in each group. Standard guidelines defined in the “Animals Bye-Laws 2008 (Scientific Procedures Issue-1) of University of Malakand” were followed [32]. Rabbits of all groups were weighed for respective dose calculation and were administered with various doses as shown in Table 4 [33]. The groups were then marked by various color coding in cages. The drugs solutions were administered per oral route using oral-gauge-tube. Group A was kept as untreated group that was fed on fresh water and food only to check whether water and food influence the blood glucose and insulin levels or otherwise.

#### 4.1.2. Experimental Animals

Adult rabbits of either sex (1.5–2.5 kg) were kept at the animal house. They were retained on 23–25 °C temperature and 45–55% relative humidity. Standard pellet diet and fresh water were used as their feed. The research studies were approved by Institutional Research Ethics Committee (IREC) under notification No: SBBU/IEC-20-01. Rabbits of all groups were exposed to light and dark cycles (12 h each cycle).

#### 4.1.3. Instruments

Blood glucose levels were determined by using Abbott’s free style glucometer. Insulin ELISA kits Accu Bind (96 wells, Monobind Inc., Lake Forest, CA, USA) were used to determine serum insulin levels. They were refrigerated at 2–8 °C. Sartorius balance was used for weighing of all drugs. Blood was collected from rabbits’ marginal ear vein through 26 Gauge disposable syringes.

#### 4.1.4. Blood Collection and Analysis

On day 1st (baseline), the 1st sample of blood was collected from all the rabbits before the administration of drugs. Accordingly, the blood was screened for the blood glucose as well as for serum insulin levels. Second blood samples were collected on day five of the drugs administration and were screened for blood glucose and serum insulin levels. The drugs were given for 5 days according to our study design. The dosage schedule for these drugs (*Moxifloxacin* and *Gemifloxacin*) is already based on its respective preclinical data at time of its approval from FDA.

Serum insulin levels were determined by using Insulin ELISA kit as per recommended techniques [34]. Briefly describing, blood was collected from marginal ear vein of the rabbits. Out of 96 well plates (Streptavidin coated), 90 for test drug and six for reference samples were used. 50 µL serum were pipetted out through micro pipette from each sample and were added to each well in plate. Then, 100 µL conjugated enzyme was added to each well and were placed in incubator for 2 h at 25 °C. After incubation time completion, all wells were washed 5 times at least with buffer saline in order to remove the unbounded proteins. 100 µL of equimolar mixture of A (Tetramethylbenzidine) and B Chromogenic were then added to each well and were placed in incubator for 15 min at normal room temperature. After completion of incubation time, stop solutions (50 µL) were added to each well and their absorbance at 450 nm were measured by ELISA reader [34].

#### 4.1.5. Histomorphological Observations

After the completion of the experiment, all the rabbits of all the groups were slaughtered and dissected to open their abdominal cavities for obtaining their pancreas. The pancreases were preserved in mixture of phosphated buffer and 10% formalin solutions. The dissected pancreatic tissues were sliced with microtome at 5 µm thickness. Hematoxylin and eosin (H&E) stains were added on each slide surface as per approved protocols [35]. The prepared slides were observed for possible histomorphological changes (beta cells cellularity and density) by using using olumphus microscope and pictures were captured [36].

### 4.2. Translating Basic Research (Pre-Clinical Studies Results) in Humans Healthy Volunteers (Clinical Trial Phase I)

#### 4.2.1. Study Procedure

Phase I clinical trial study was conducted at single center (Department of Pharmacy Shaheed Benazir Bhutto University, Sheringal Dir Upper Khyber Pakhtunkhwa, Pakistan) according to good clinical practice guidelines. One group received *Moxifloxacin* and another group received *Gemifloxacin*. *Moxifloxacin* and *Gemifloxacin* were of standard pharmaceutical firms (Getz healthcare Pharmaceutical, Karachi and Sami Pharmaceutical Karachi, respectively). *Moxifloxacin* (400 mg) and *Gemifloxacin* (320 mg) were used in once daily dosing by participants of respective groups for 5 days as per Table 5. Blood samples were collected on day one (baseline) and on day five and were analyzed for fasting blood glucose and serum insulin levels.

Both male and female volunteers of age range from 20 to 40 years were eligible for the study. Body weights, basal mass index, and other important parameters, such as(oral body temperatures, blood pressure, and pulse rates) were appropriately screened for all participants. They were informed and consents were signed from all participants. Lactating and pregnant women and subjects whose age limit <20 years or >40 years were excluded. Similarly, subjects having QTc ≥ 450 ms, having diabetes mellitus history or family history, facing hypokalemia or cardiovascular diseases, or with a predetermined history of fluoroquinolones allergy were also excluded from trial. The subjects participating in the clinical trial were instructed to use a scheduled uniform diet and were informed not to use any other medicine(s) during trial period. A total of 65 participants were screened out of which 5 were not meeting the eligibility. They were equally randomized into two groups on simple random sampling technique using R-statistical software in order to decrease the chances of biasness. The study protocols were approved by institutional Research Ethics Committee of Shaheed Benazir Bhutto University Sheringal Dir Upper notified via notification SBBU/IEC-20-01 and was also registered with government agency for clinical trial registration. Clinical Trials.gov identifier: NCT04692623.

#### 4.2.2. Collection of Blood for Blood Glucose and Serum Insulin Levels

On day 1st (baseline), the 1st blood samples (3–5 mL) were collected from peripheral vein of all treated volunteers of both groups and were assessed for blood glucose and serum insulin levels. The second blood samples were collected on day five for determination of blood glucose and serum insulin, respectively. The sites of veins were properly sterilized with dettol and sterilized cotton to prevent vein site from chances of infections. Insulin ELISA kits (Accuband) manufactured by Monobind Inc., Lake Forest, CA, USA) were used for determination of insulin level [34], as per procedures mentioned above in Section 4.1.4 [34].

### 4.3. Statistical Analysis

R-Statistical software 3.1.3 was used for the unbiased randomization of all participants into two equal groups. Mean blood glucose and insulin values on their respective baseline and on day five were plotted using Graph Pad Prism version-6 (Graph Pad Prism, San Diego, CA, USA). The changes in baseline blood glucose and serum insulin levels of all treated groups were compared using one way ANOVA (*p* < 0.05). One-way (ANOVA) tests were followed by Wilcoxon signed rank test to check the statistical difference.

## 5. Limitations

In our present study, we only determined the effect of these drugs on the blood glucose levels via any surge in the serum insulin level. Severe hypoglycemia was not reported either in the rabbits nor in the healthy volunteers. This may involve multiple compounding factors and underlying mechanisms, such as the secondary messenger system via glucagon and epinephrine release and cortisol and growth hormone release that counteract hypoglycemia. Therefore, further studies are required to check the underlying mechanisms of this insulinotropic phenomenology of *Moxifloxacin* and *Gemifloxacin*.

## 6. Conclusions

It is concluded that *Moxifloxacin* and *Gemifloxacin* significantly increased the secretion of insulin, which subsequently lowered the blood glucose in a working pancreas both in experimental animals and human healthy volunteers.

## 7. Recommendations

The clinicians shall use *Moxifloxacin* and *Gemifloxacin* with caution in controlled maturity onset diabetic patients (working pancreas) already using antidiabetic drugs. The use of *Moxifloxacin* and *Gemifloxacin* may be avoided in maturity onset diabetic patients to avoid the possible risk of hypoglycemia. It is best advised to clinicians to opt for an alternate mode of antibiotic therapy in such diabetic patients.

## Figures and Tables

**Table 1:** *Moxifloxacin*, *Gemifloxacin*, and standard *Glibenclamide* effects on blood glucose levels using in vivo rabbits model study.

Descriptions (Groups)	Baseline (Day 1st) Glucose (mg/dL) (Mean ± SD)	Day 5th Glucose (mg/dL) (Mean ± SD)	*p*-Value
Untreated	108.8 ± 16.7	116.5 ± 8.6	0.3402 ^ns^
*Moxifloxacin*	112 ± 4.5	86.17 ± 11.2	0.0002 ***
*Gemifloxacin*	117.3 ± 7.2	93.5 ± 7.4	0.0122 **
*Glibenclamide*	121.17 ± 16.17	89.7 ± 9.6	0.0011 ***

*p*-values show the level of significance compared to their respective day 1st (baseline) on day 5th values of blood glucose levels. *p*-values ˂ 0.0001 and ˂0.05 were expressed as *** and ** respectively. ns stands for non-significant. Untreated means they were fed only water and food and were kept as negative control group. *Glibenclamide* was given to positive control group.

**Table 2:** *Moxifloxacin*, *Gemifloxacin*, and standard *Glibenclamide* effects on serum insulin levels using in vivo rabbits model study.

Description (Group)	(Day 1st) Insulin (µIU/mL)	(Day 5th) Insulin (µIU/mL)	*p*-Value
Untreated	3.3 ± 0.44	3.28 ± 0.16	0.06 ^ns^
*Moxifloxacin*	4.09 ± 0.92	52.45 ± 10.7	<0.0001 ****
*Gemifloxacin*	4.50 ± 1.37	23.48 ± 12.70	0.0088 ***
*Glibenclamide*	4.43 ± 1.95	68.13 ± 14.40	<0.0001 ****

*p*-values show the level of significance on day 5th to their respective day 1st (baseline) insulin values. ns stands for non-significant. *p*-values ˂ 0.0001 and ˂0.05 were expressed as **** and *** respectively. Untreated means they were fed only water and food and were kept as negative control group. *Glibenclamide* was given to positive control group.

**Table 3:** Demographic information and baseline clinical characteristics of the study group participants in the clinical trial study.

Items	*Moxifloxacin* Group (*n* = 25) (Mean ± SD)	*Gemifloxacin* Group (*n* = 25) (Mean ± SD)
Age (Years)	27.4 ± 2.9	31.4 ± 5.4
Gender, *n* (%)	Male 21 (84%)	Male 22 (88%)
	Female 04 (16%)	Female 03 (12%)
Weight (Kg)	68.16 ± 5.1	67.4 ± 4.1
Body Mass Index (BMI, Kg/m^2^)	23.9 ± 1.7	24.0 ± 1.9
Systolic Blood Pressure (mm Hg)	120.8 ± 10	116.8 ± 7
Diastolic Blood pressure (mm Hg)	79.6 ± 4.5	78.8 ± 3.3
Pulse Rate (BPM)	77.4 ± 5.7	78.4 ± 6.7
Body Temperature (°C)	37.1 ± 0.3	37 ± 0.3
DM History/Familial History *n* (%)	Yes 0 (0%)	Yes 0 (0%)
	No 25 (100%)	No 25 (100%)
Smoking Status, *n* (%)	Yes 0 (0%)	Yes 0 (0%)
	No 25 (100%)	No 25 (100%)
Allergy to drugs/food/other substances, *n* (%)	Yes 0 (0%)	Yes 0 (0%)
	No 25 (100%)	No 25 (100%)

**Table 4:** Doses and dosage schedule of the groups in in vivo rabbits model study.

Groups	Drugs	Doses and Dosages PO
Group A	Untreated	Pellet food and fresh water only
Group B	*Moxifloxacin*	5 mg/kg/day once daily.
Group C	*Gemifloxacin*	5 mg/kg/day once daily.
Group D	*Glibenclamide*	0.2 mg/kg/day once daily.

**Table 5:** Doses and dosage schedule of the groups in the clinical trial.

Groups	Drugs	Doses and Dosages	Route
Group A	*Moxifloxacin (M)*	400 mg once daily.	Orally (PO)
Group B	*Gemifloxacin (G)*	320 mg once daily.	Orally (PO)

## Data Availability

The datasets (histological photographs) for the current study are available from the corresponding authors on reasonable request.

## Abbreviations

ATP: Adenosine Tri Phosphate, ADP: Adenosine Di Phospahte, BP: British Pharmacopia, CADRMP: Canadian Adverse Drug Reaction Monitoring Program, ELISA: Enzyme Linked Immunosorbent Assay, FQs: Fluoroquinolones, FDA: Food and Drug Administration, >: Greater, <:Less, H&E: Heamatoxylin and eosin, mg: Milligram, µM: Micromolar, µL:Microliter, PO: Per oral, Kg: Kilogram, KATP: ATP-Sensitive potassium channels, SUR1 and SUR2:Sulfonyl Urea Receptors1,2, VG: Voltage Gated, WFI: Water for injection.
